# Fatal Non-aneurysmal Subarachnoid Haemorrhage During Bridge-to-Transplant Rehabilitation in a Patient With Biventricular Assist Devices

**DOI:** 10.7759/cureus.117013

**Published:** 2026-09-27

**Authors:** Christopher Youssef

**Affiliations:** 1 Medicine, University of Cambridge, Cambridge, GBR

**Keywords:** acquired von willebrand syndrome, bivad, heparin induced thrombocytopenia (hit), left ventricular assist device(lvad), spontaneous subarachnoid hemorrhage

## Abstract

Subarachnoid haemorrhage (SAH) is a rare but catastrophic complication in patients supported by biventricular assist devices (BiVADs). While mechanical circulatory support (MCS) is a life-saving bridge to transplantation, it introduces complex haemostatic challenges.

We report the case of a 32-year-old male with hypertrophic cardiomyopathy who developed cardiogenic shock. He was bridged from central veno-arterial extracorporeal membrane oxygenation (cVA-ECMO) to BiVAD support. His clinical course was complicated by suspected heparin-induced thrombocytopaenia (HIT), but further testing proved he was HIT negative. After weeks of haemodynamic stability and the commencement of physical rehabilitation for heart transplant listing, the patient suffered a sudden and profound neurological decline. Imaging revealed a diffuse, non-aneurysmal SAH occupying the basal cisterns. Neurosurgical intervention included placement of an external ventricular drain; however, he further declined, and support was withdrawn.

This case highlights the challenging haemostatic landscape inherent to ventricular assist devices (VADs), where standard laboratory tests fail to reflect profound functional coagulopathy. Ultimately, this case serves as a critical reminder that BiVADs create a challenging haemostatic environment that demands more sophisticated solutions.

## Introduction

Subarachnoid haemorrhages (SAH) are defined as bleeding into the subarachnoid space, the area between the arachnoid and pia mater, and are most commonly associated with aneurysmal rupture [1]. While aneurysmal SAH is a well-recognized entity in the general population, non-aneurysmal SAH represents a distinct and devastating subset of ventricular assist device (VAD) patients, often driven by device-induced coagulopathy rather than structural vascular defects. Up to 10.6% of left ventricular assist device (LVAD) patients have intracranial haemorrhages, with 23.6% of these being SAH [2]. Here we present a case of a young gentleman, admitted with cardiogenic shock, placed on a biventricular assist device (BiVAD), who has suffered a severe form of this rare complication.

## Case presentation

Initial presentation and history

A 32-year-old male of African ethnicity with a history of end-stage hypertrophic cardiomyopathy (LVEF of 30-35%) was referred to our hospital in acute decompensated heart failure. His past medical history included paroxysmal atrial fibrillation, type 2 diabetes mellitus, asthma, hepatic steatosis and CKD stage 3a [3]. On admission, he exhibited severe respiratory distress with severe shortness of breath, peripheral oedema and a respiratory rate of 30. His initial blood tests showed an NT-proBNP of 16,000 pg/mL (normal <400 pg/mL) and signs of hepatic congestion.

Clinical course

Worsening pulmonary oedema led to escalation to intensive care, where he quickly deteriorated further into cardiogenic shock. This was characterised by increasing requirements of vasoactive agents, lactate peaking at 18 mmol/L (0.5-2.2mmol/L) and a SvO_2_ of 44% (60-80%). Therefore, he was established on central veno-arterial ECMO before transitioning to a CentriMag biventricular assist device.

In the following two weeks, the patient’s cardiovascular stability improved, including weaning from inotropes and commencement of rehabilitation. He had notable complications during this period of stability. This included an acute kidney injury, requiring filtration and thrombocytopaenia. Suspicion for heparin-induced thrombocytopaenia (high 4Ts score of 6) led to a transition to argatroban, but following a negative platelet factor 4 (PF4) enzyme-linked immunosorbent assay (ELISA) and a partial recovery of his platelets, he was restarted on heparin within a week.

Neurological event and radiology

The patient suffered a sudden reduction in GCS to E1V1M1, with his right pupil being fixed and dilated at 5mm. At the time, his platelet count was 130 x10^9^/L (150-450 x10^9^/L) and his clotting parameters were within the therapeutic anticoagulation range (aPTT ratio of 1.6, target 1.5-2.0) (Figures 1-2). The renal and liver function parameters are shown in Appendix A and Appendix B.

**Figure 1:**
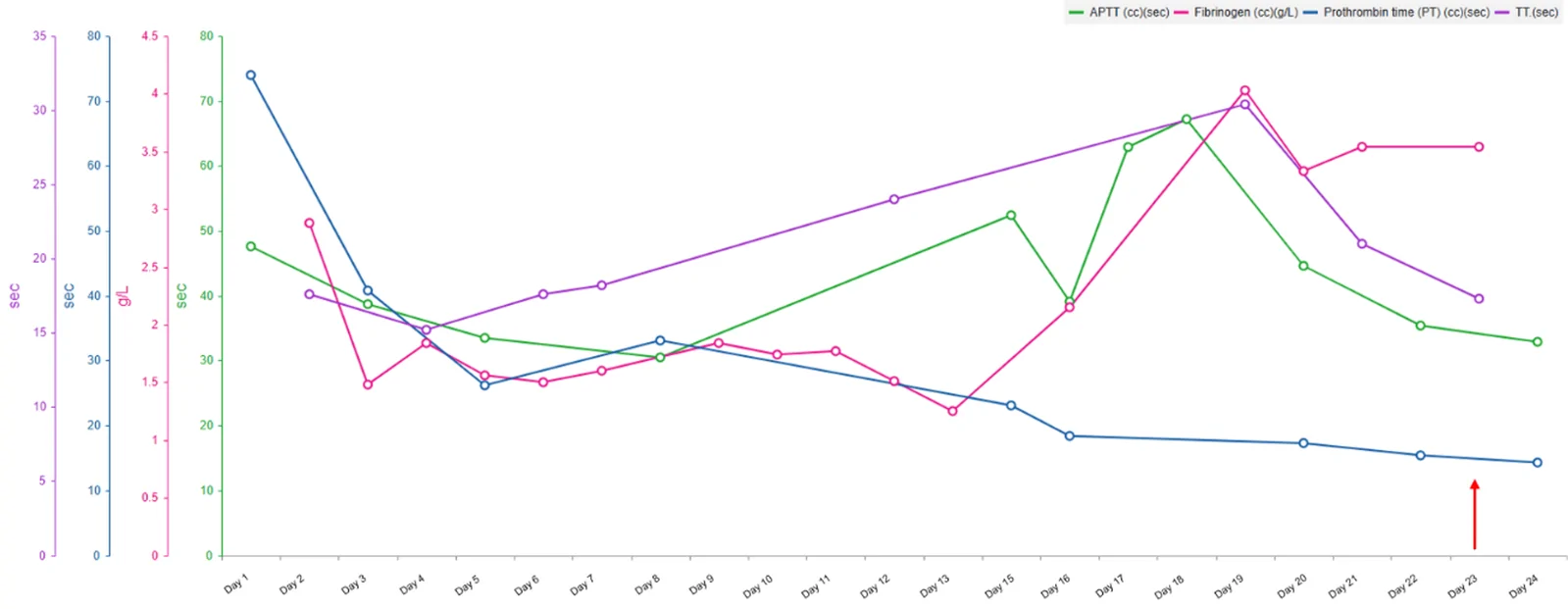
Coagulation lab parameters from admission to intensive care to day of neurological deterioration. Arrow pointing at the day of neurological deterioration.

**Figure 2:**
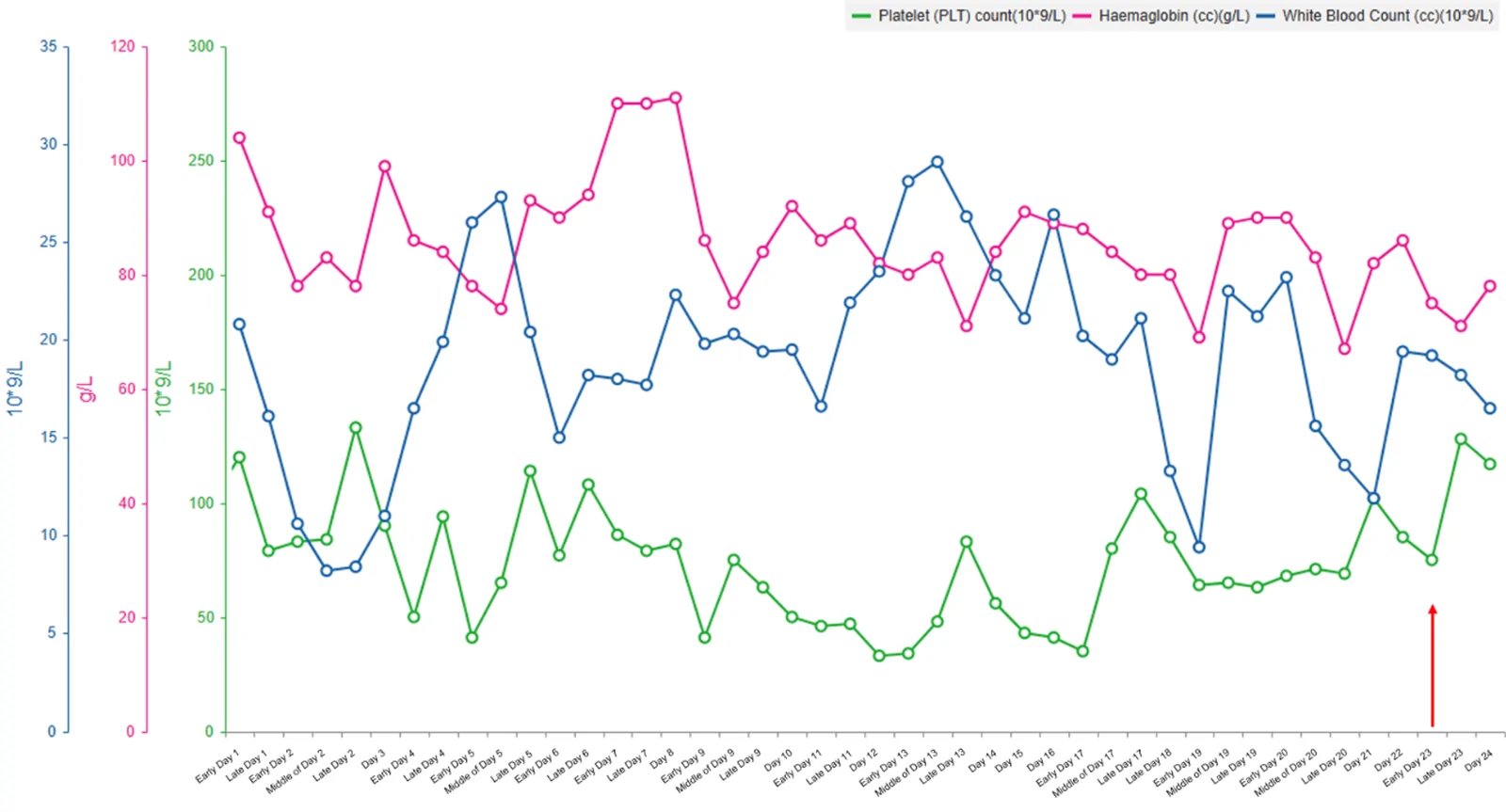
Graph showing lab parameters, with admission to intensive care and neurological event as demonstrated by arrow. Arrow pointing at the day of neurological deterioration.

Urgent CT imaging revealed a large acute subarachnoid haemorrhage occupying the basal cisterns and posterior fossa, with extension into the fourth ventricle. CT angiography (CTA) demonstrated no underlying aneurysm or major vessel occlusion (Figure 3).

**Figure 3:**
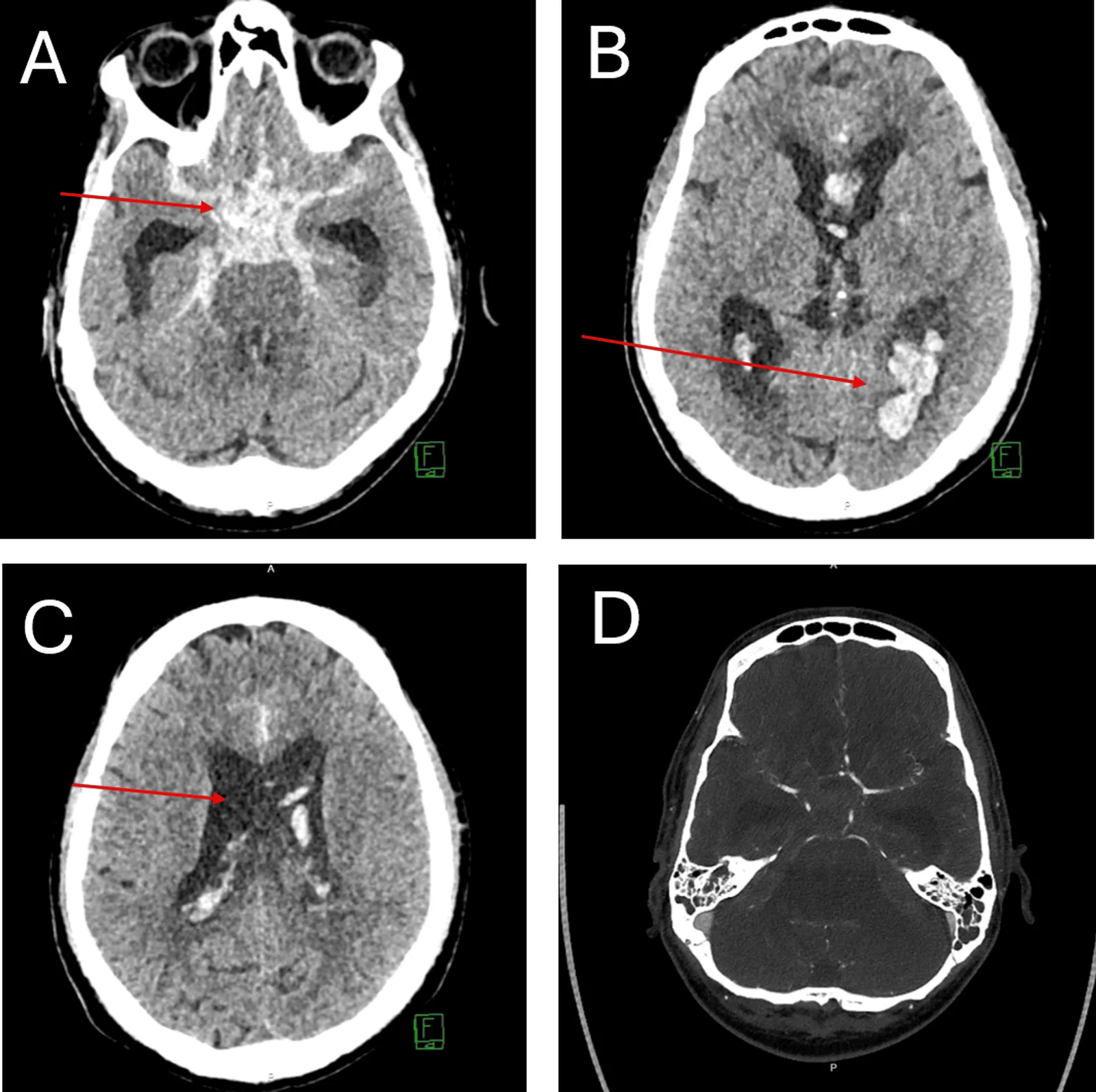
A CT head showing a large subarachnoid haemorrhage. (A) Shows blood occupying the basal cisterns and Sylvian fissure; (B) demonstrates blood within the occipital horn of the lateral ventricle; (C) demonstrates a dilated ventricle system; (D) demonstrates a CT angiogram with an unremarkable Circle of Willis and no aneurysms demonstrated. The arrow in A points to blood within the basal cisterns and tracking into the Sylvian fissure. The arrow in B points at blood within the occipital horn of the lateral ventricle. The arrow in C shows that the lateral ventricle is dilated.

Surgical intervention

With a confirmed intracranial haemorrhage, the patient's heparin infusion was held, but no reversal agent was given due to concern of catastrophic clotting within the BiVAD circuitry. Furthermore, with support from our neurosurgical team, a right frontal burr hole was performed. Upon dural opening, a first-pass catheterisation yielded high-pressure CSF. A right frontal external ventricular drain was placed with good xanthochromic flow. Despite this, there was further neurological deterioration in the following hours. This involved a worsening respiratory pattern, pupils becoming fixed and dilated bilaterally, and increasing haemodynamic support requirements. In light of the poor prognosis, support was withdrawn in consultation with the family. Table 1 shows a timeline of events from initial deterioration to this event, highlighting changes in anticoagulation methods. 

**Table 1:** Timeline of events, including changes to anticoagulation titration. VA-ECMO: veno-arterial extracorporeal membrane oxygenation; ECMO: extracorporeal membrane oxygenation; BiVAD: biventricular assist device; HIT: heparin-induced thrombocytopenia; APR: activated partial thromboplastin time ratio; CT: computed tomography; CTA-negative: computed tomography angiography-negative; EVD: external ventricular drain; SAH: subarachnoid haemorrhage; LVEF: left ventricular ejection fraction; SvO₂: mixed venous oxygen saturation; GCS: Glasgow Coma Scale; CSF: cerebrospinal fluid.

Date	Events and anticoagulation status
Day 1	Admitted to intensive care with severe heart failure, escalated to VA-ECMO. Vitamin K and prothrombin complex concentrate were given to reverse prior warfarin dosing.
Day 2	Bleeding from ECMO cannulas + nasal bleeding, managed with a balloon.
Day 5	Converted to BiVAD; bleeding stopped. Patient is established on heparin with an APR target of 1.5-1.8
Day 7	Sedation is held; recovering neurology.
Day 8	Persistently low platelets; HIT screen sent, argatroban started at 0.5 mcg/kg/min and reduced by half due to nasal bleeding
Day 9	Patient is fully conscious; begins rehab
Day 12	Pleural effusions are drained in theatre
Day 15	HIT screen negative; heparin restarted; tightened APR target of 1.2-1.5
Day 22	Trial off mechanical ventilation, rehabing well
Day 23	Asymmetric pupils and deterioration in neurology prompted a CT head, demonstrating a CTA-negative SAH. EVD inserted at bedside. Further worsening neurology throughout the day, with family counselled
Day 24	Patient passes away; support removed

## Discussion

This case describes a poorly characterised and serious complication of BiVAD devices in a patient with increasingly complex anticoagulation. As with all forms of mechanical circulatory support, there is an increased risk of bleeding, but this must be balanced against the risk of catastrophic clot-induced device failure.

Sudden neurological decline in patients on BiVADs presents a narrow range of differentials. Although the CT angiogram showed no saccular dilation or focal vascular irregularities, digital subtraction angiography remains vital to rule out macrovascular events, though it was deferred due to rapid neurological deterioration. The team suspected microvascular, rather than macrovascular, friability as the primary cause.

In BiVADs, there is a loss of cyclical shear stress due to their inherently narrowed pulse pressure. The effect of this on cerebral arterial walls has been demonstrated in vivo to promote endothelial dysfunction and impair angiogenesis [4]. This endothelial damage and impaired angiogenesis have been suggested to trigger the formation of undetectable micro-aneurysms, which may cause intracerebral bleeds [5].

In addition, the heightened risk for bleeding is thought to be related to a range of aetiologies, including acquired von Willebrand Syndrome (AvWS) and platelet dysfunction. In AvWS, high shear stress within the pump causes mechanical and ADAMTS13-mediated enzymatic breakdown of large von Willebrand factor multimers, which prevents platelets from adhering to one another and forming a stable plug [6]. This is further compounded by platelet dysfunction, where high shear stress promotes the chronic externalisation of P-selectin and the initial activation of glycoprotein Ib receptors; however, this persistent mechanical overstimulation ultimately triggers the proteolytic shedding and downregulation of these surface markers, effectively neutralising the platelet’s capacity to respond to prothrombotic signals [7]. Whilst it is true that this patient had a normal platelet count at deterioration, these platelets were likely highly dysfunctional, as demonstrated by thromboelastography. 

These factors, compounded by hepatic congestion, create a challenging haemostatic landscape in patients with a higher risk for both catastrophic thrombosis and a higher propensity for devastating intracranial bleeds. Currently, as no international guidelines exist, there is substantial variability in anticoagulation protocols amongst transplant centres [8]. Importantly, this means that there is equally varied use of thromboelastography, a form of platelet function testing that is proving vital in MCS use. Overall, the future promise of AvWS treatment [9] and modified device design [10] produces optimism in reducing these devastating complications.

## Conclusions

This case illustrates a catastrophic complication of BiVAD in a young, rehabilitating patient. The development of a large subarachnoid haemorrhage despite an improving platelet count and a stable aPTT ratio highlights the profound difficulty in managing haemostasis in the setting of BiVAD and hepatic injury. The absence of an underlying aneurysm hints that this bleed was possibly a consequence of complex functional coagulopathy that is inherent to MCS. This case adds to the limited BiVAD-specific literature on non-aneurysmal subarachnoid haemorrhages and serves as a critical reminder that clinical stability during rehabilitation can be interrupted by rapid-onset intracranial events. Finally, it underscores the necessity for more robust strategies to monitor functional haemostasis in this patient group and for further research into BiVAD-specific risks.

## Figures and Tables

**Figure 4:**
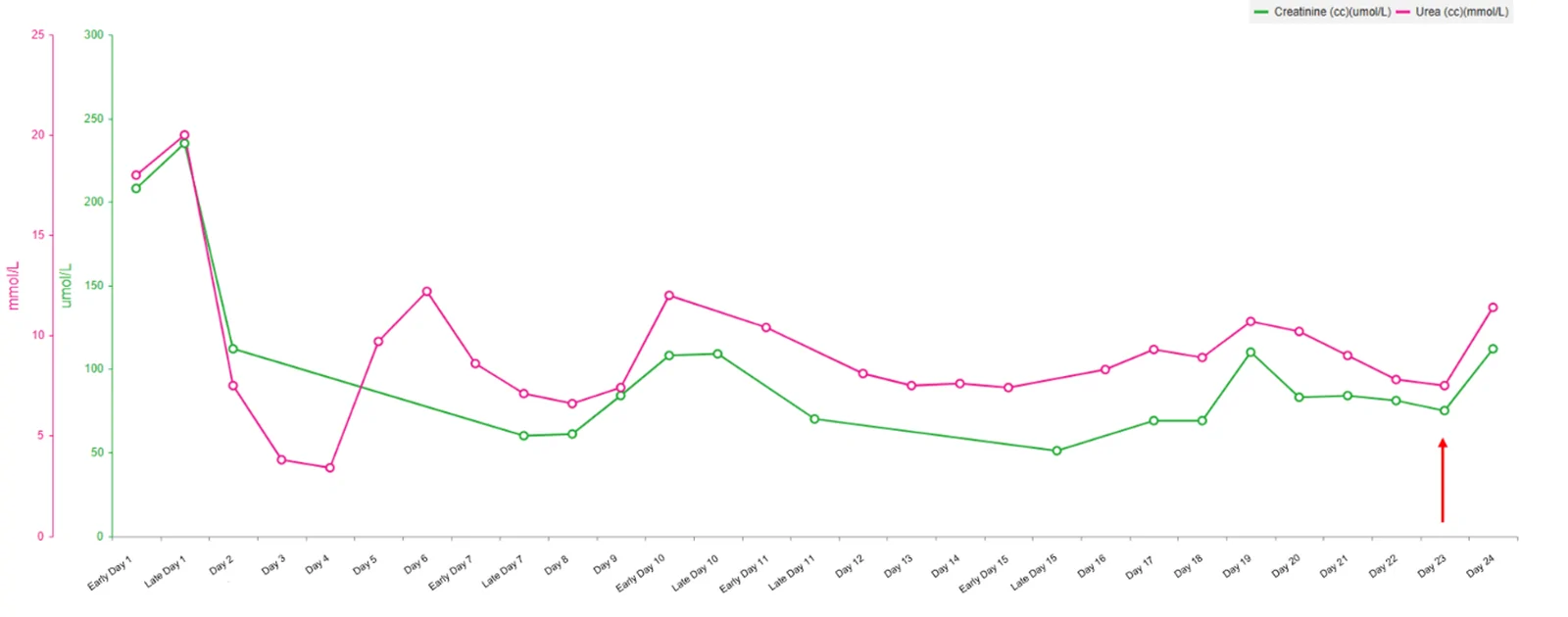
Renal function throughout admission. Arrow indicates the day of neurological deterioration.

**Figure 5:**
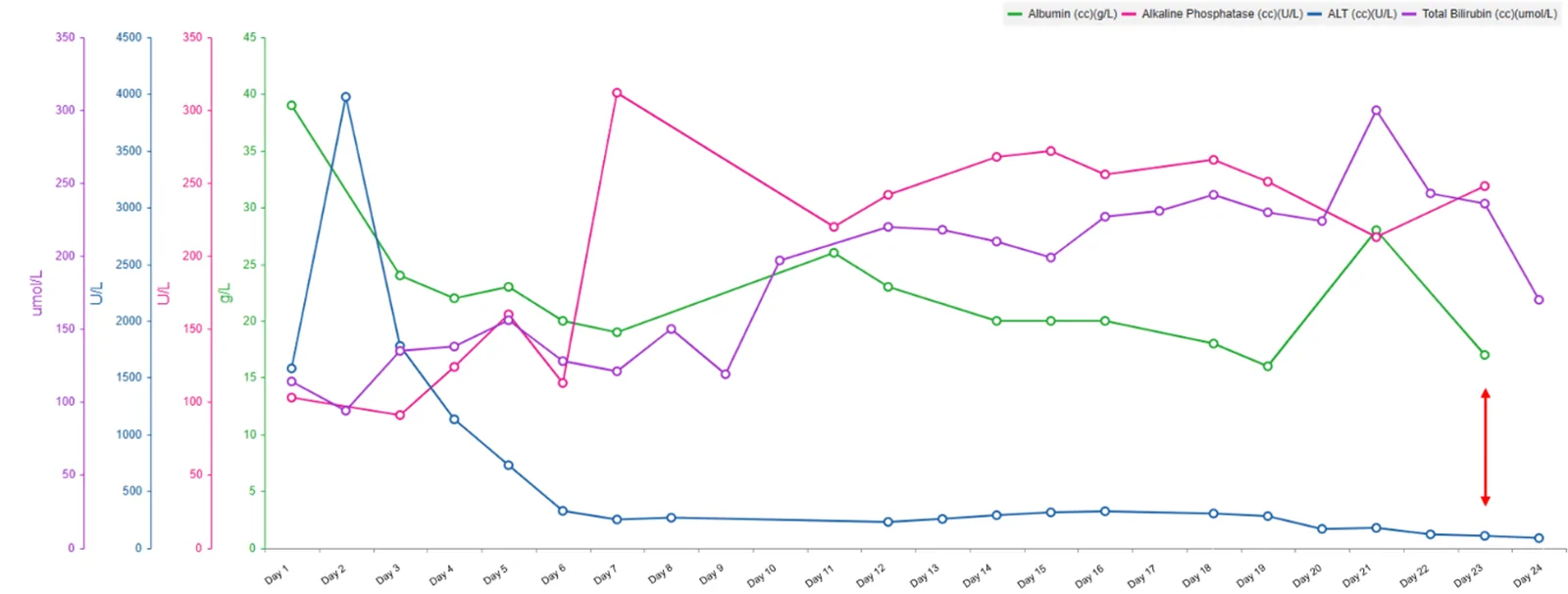
Liver function lab parameters during admission. Arrow indicates the day of neurological deterioration.

## Appendices

Appendix A

Graph showing lab parameter trends for renal function.

 Appendix B

Graph showing lab parameter trends for liver function.
